# Supplementary material for: Freeze-frame imaging of synaptic activity using SynTagMA
Source: Nat Commun. 2020 May 18;11:2464. doi: 10.1038/s41467-020-16315-4 (PMC7235013; doi:10.1038/s41467-020-16315-4)
Supplement: Supplementary file 1 — Supplementary Information [file 41467_2020_16315_MOESM1_ESM.docx]

**Supplementary Information**

**Freeze-frame imaging of synaptic activity using SynTagMA**

Perez-Alvarez A., Fearey B.C., O’Toole R.J., Yang W., Arganda-Carreras I., Lamothe-Molina P.J., Moeyaert B., Mohr M.A., Panzera L.C., Schulze C., Schreiter E.R., Wiegert J.S., Gee C.E., Hoppa M.B., Oertner T.G.


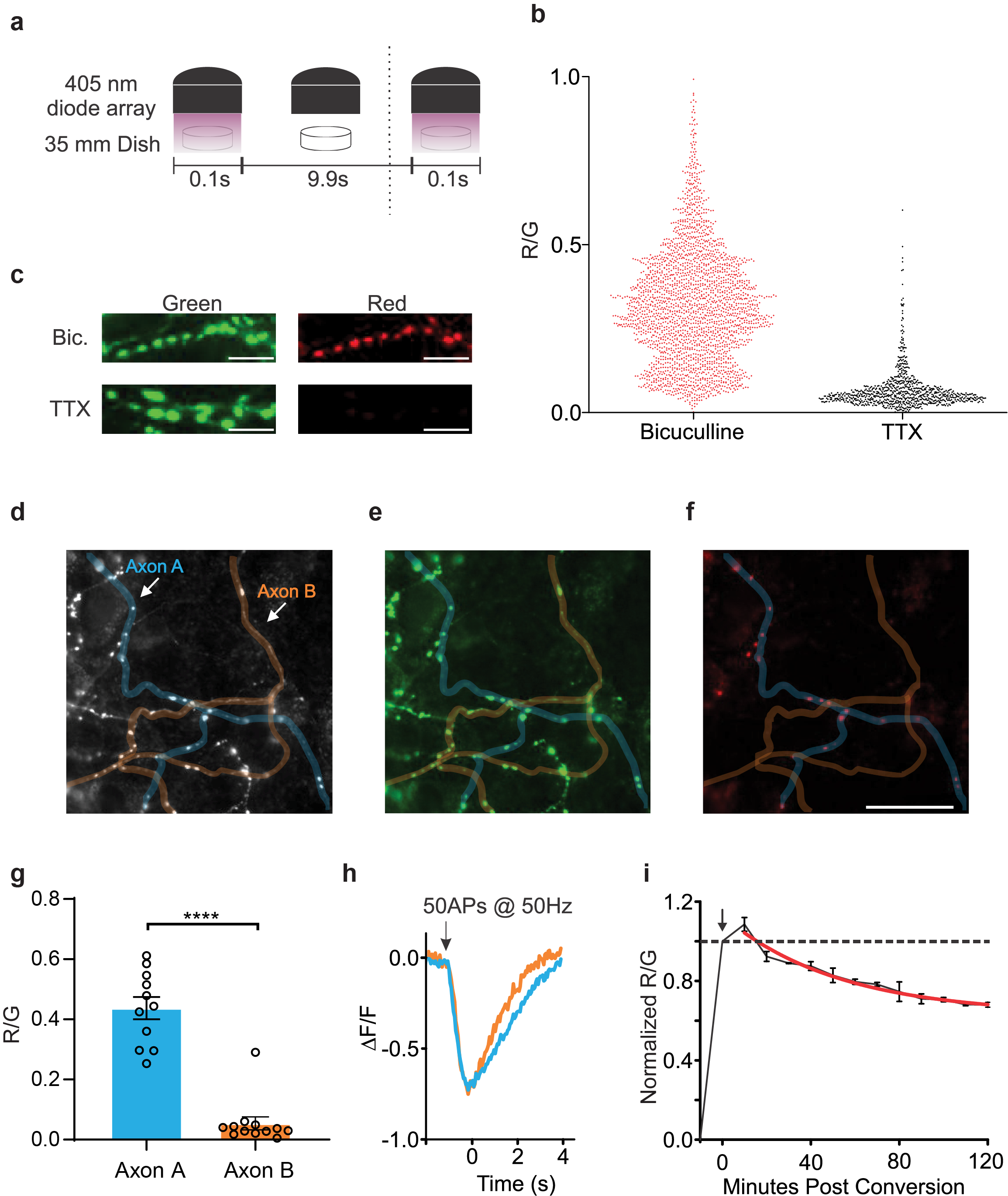


**Supplementary Figure 1: Spontaneous circuit activity of cultured primary hippocampal neurons drives photoconversion of preSynTagMA**.**(a)** LED arrays (51 diodes, 405 nm) were placed in an incubator (5% CO_2_, 37°C) and set to flash for 100 ms every 10 s for 4 hours. LED arrays were placed on top of 35 mm dishes containing cultured primary hippocampal neurons. **(b)** Example green and red raw fluorescence of boutons when incubated with bicuculline to increase activity or TTX to block activity while illuminating with violet light as shown in **a**.**(c)** R/G ratio of individual boutons with bicuculline (n = 2456 synapses, 3 experiments) or TTX (n = 866 synapses, 4 experiments) **(d)** Axons from two neurons in bicuculline condition (axon A, blue overlay; axon B, orange overlay). **(e)** Raw green fluorescence. **(f)** Raw red fluorescence. Note that photoconversion (red fluorescence) was restricted to boutons of axon A. **(g)** Mean R/G ± SE from boutons of axon A (n = 11 boutons, R/G = 0.44 ± 0.04) and axon B (n = 12 boutons, R/G = 0.05 ± 0.02) (****p < 0.0001, Student’s t-test, unpaired), indicating differential activity. **(h)** Dimming of green fluorescence from axons A (blue) and B (orange) during a 50 AP, 50 Hz stimulus train. Similar dimming indicates both axons could fire action potentials and had similar calcium influx during induced spiking. Therefore, the lack of photoconversion in bicuculline was most likely due to a lack of spiking in axon B. **(i)** Slow decay of R/G ratio following strong photoconversion (40 x 1 s light, 405 nm, paired exactly with 20 APs @ 50Hz). Images were acquired every ten minutes for two hours (n = 4 cells). Data are shown as mean ± SEM. Scale bars: 5 µm (c), 50 µm (d-f)**.**


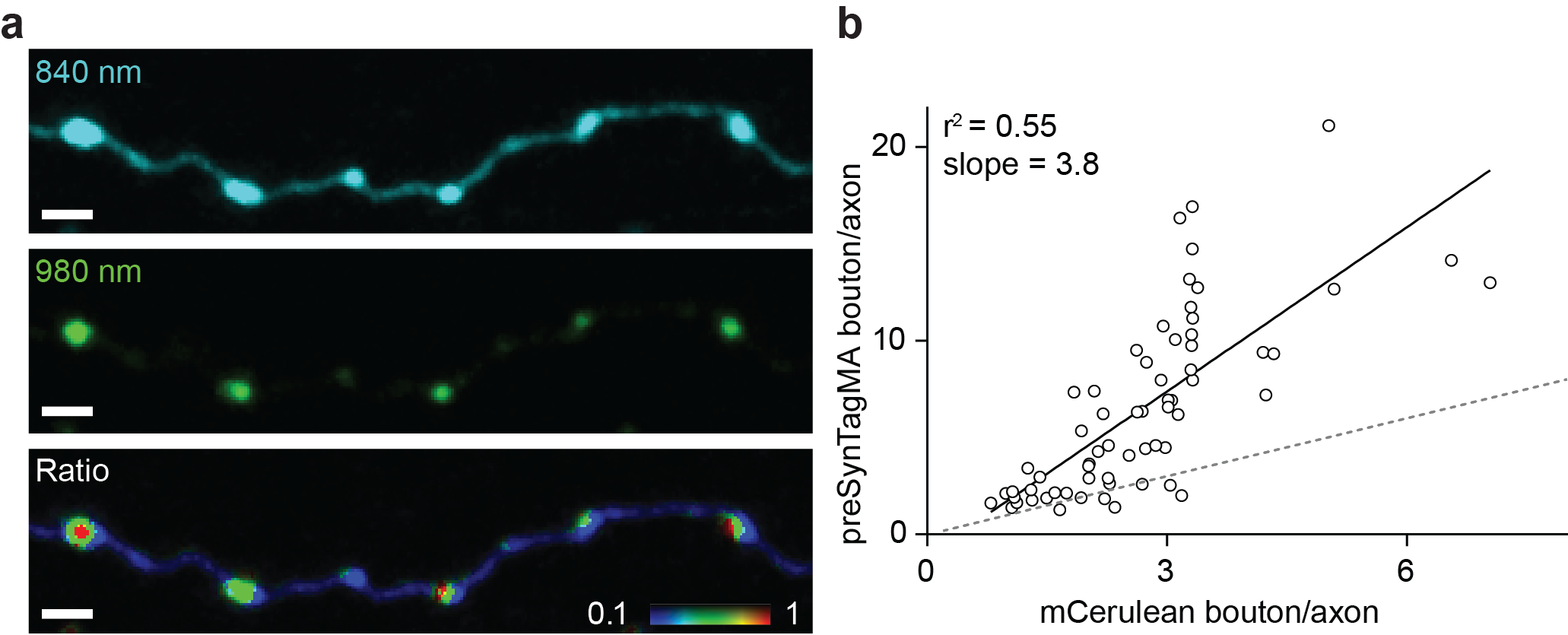


**Supplementary Figure 2: Enrichment of preSynTagMA in Schaffer collateral boutons. (a)** Two-photon image of hippocampal slice culture showing axonal boutons in *stratum radiatum*, originating from CA3 neurons expressing preSynTagMA and mCerulean as cytosolic filler. Upper panel: mCerulean (840 nm), middle panel: preSynTagMA (980 nm), lower panel: green/cyan ratio image, indicating labeled vesicle clusters. Scale bars: 2 µm. **(b)** The ratio of preSynTagMA bouton to axonal shaft fluorescence vs the ratio of mCerulean bouton to axon fluorescence for n = 64 boutons. Solid line: linear fit. Dashed line (slope = 1) indicates the expected location of boutons without synaptic vesicles. Experiment was reproduced in two slices.


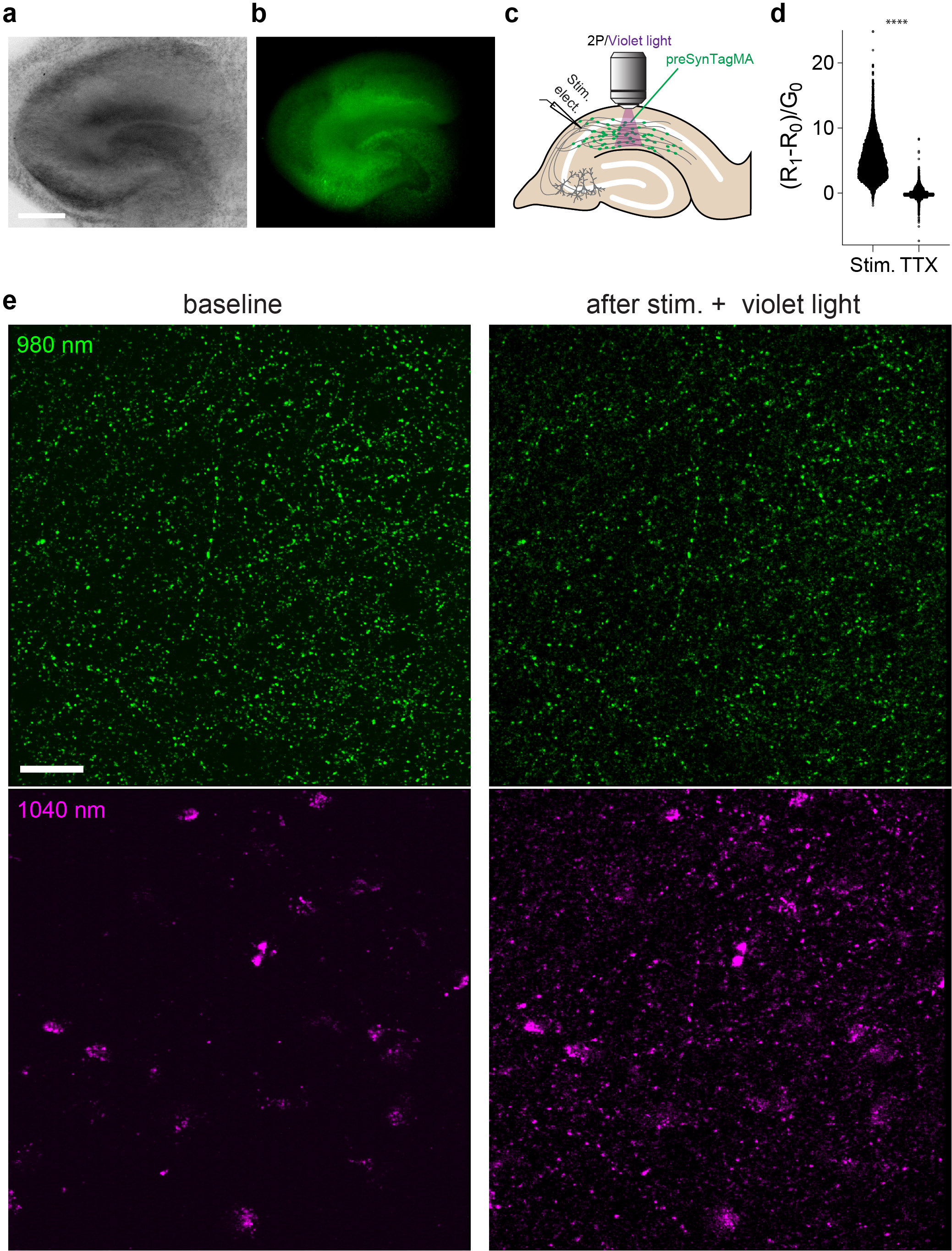


**
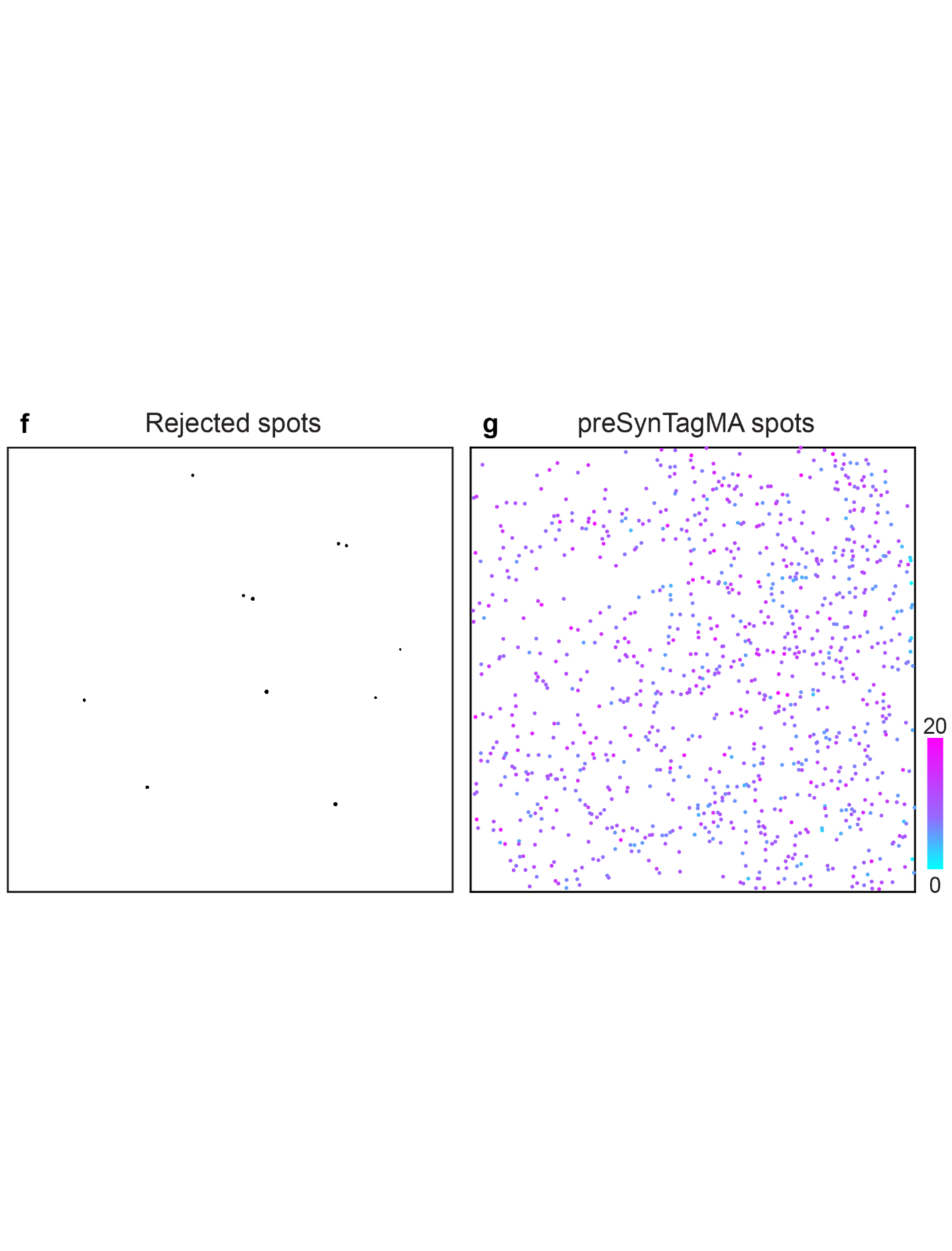
**

**Supplementary Figure 3: Detection of active boutons in rat hippocampal slices. (a)** Bright field image of a rat organotypic hippocampal slice microinjected with AAV2/9-syn-preSynTagMA in the CA3 area. **(b)** Wide-field fluorescent image showing preSynTagMA signal. **(c)** Scheme showing stimulus electrode and imaging area in *stratum radiatum*. Axon fibers were either stimulated with a glass monopolar electrode or silenced with TTX (1 μM). The electrode tip was placed in *stratum radiatum* to deliver an electrical stimulus to Schaffer collateral axons (0.2 ms pulses at 50Hz during 500 ms). Violet light (200 ms, 16 mW mm^-2^) was delivered with a 1 s delay relative to stimulus. This paired protocol was repeated 25 times at 0.1 Hz. **(d)** Violin plot of bouton conversion [(R_1_ – R_0_/G_0_)] in stimulated (5.13 ± 0.02; n= 12887 boutons, 5 slices) vs TTX groups (0.02 ± 0.01; n = 10193 boutons, 3 slices). A Mann Whitney test showed stimulated vs. TTX groups were significantly different (****p<0.0001). **(e)** Two-photon z-stacks before (green, acquired at 980 nm) and after (red, acquired at 1040 nm) the electrical stimulus protocol paired with violet light was repeatedly applied. **(f)** Automated analysis using SynapseLocator. Green spots with high red fluorescence at baseline (R_0_) were rejected. **(g)** Analysis of all spots with low R_0._ Scale bars: 500 µm (a,b), 25 µm (e-g).

**
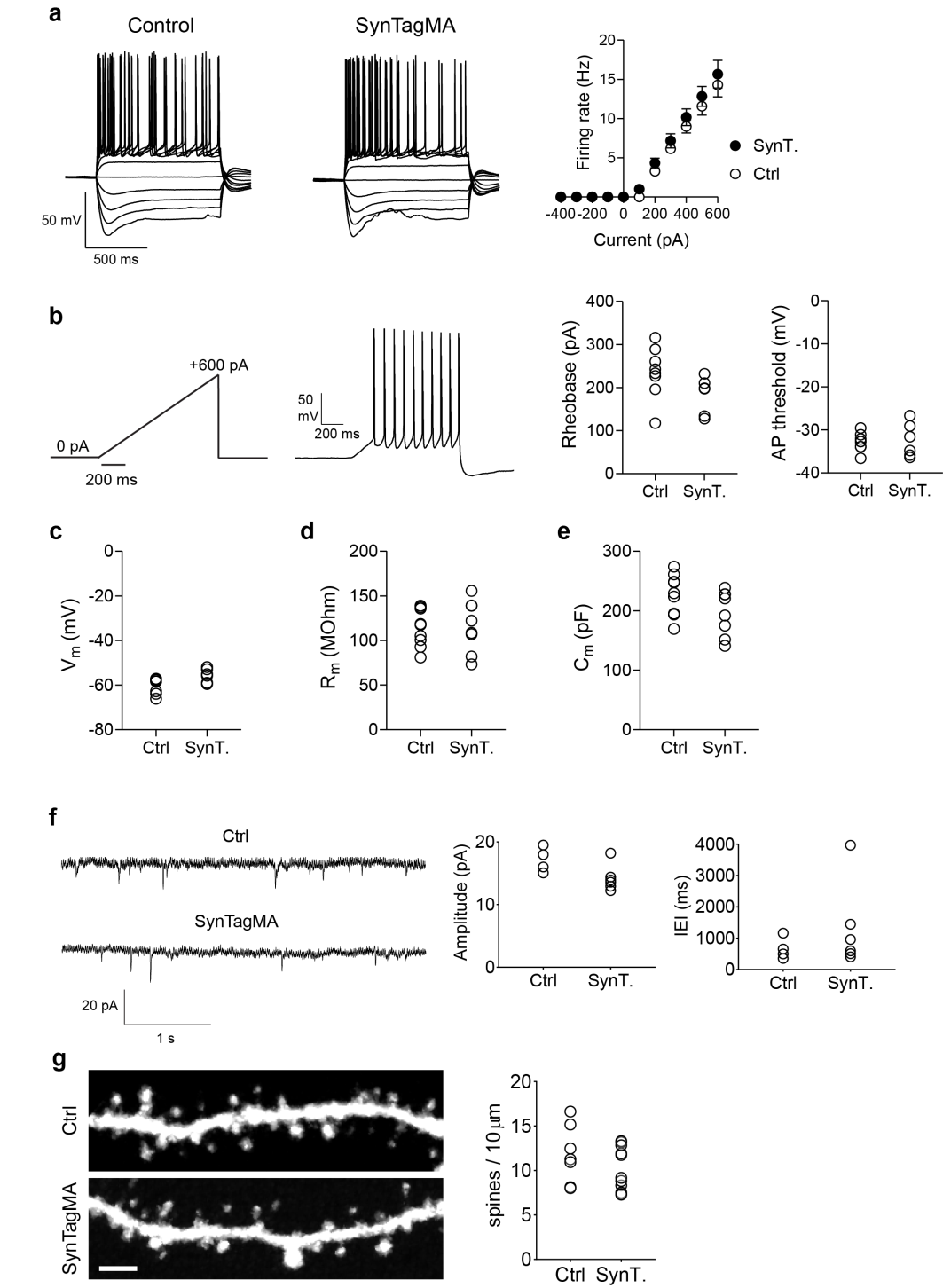
**

**Supplementary Figure 4: Properties of SynTagMA-expressing neurons**. **(a)** *Left,* example of voltage responses of CA1 pyramidal neurons expressing either mCerulean (Ctrl, n = 6 neurons, 6 slices) or mCerulean and postSynTagMA (SynT., n = 7 neurons, 7 slices) elicited by current steps from -400 pA to +600 pA (1 s duration, 0.067 Hz). *Right,* plot showing mean firing rate during voltage steps (mean ± SEM). **(b)** Current ramp (1 s duration from 0 to +600 pA) and example response of a SynTagMA-expressing neuron used to determine the rheobase and action potential (AP) threshold. Rheobase of SynTagMA-expressing neurons was not different from mCerulean controls (Ctrl: 235.2 ± 21.4 pA, n = 8 neurons; SynT: 183.2 ± 17.4 pA, n = 6; p = 0.108, Mann-Whitney test). AP threshold of SynTagMA-expressing neurons was not different from mCerulean controls (Ctrl: -32.8 ± 0.74 mV, n = 8 neurons; SynT: -32.4 ± 1.6 mV, n = 6, p = 0.80, two-tailed Student’s t test). **(c)** Resting membrane potential (Vm, Ctrl: -59.9 ± 1.1 mV, n = 9 neurons; SynT: -55.7 ± 1.3 mV, n = 6; p = 0.088, Mann-Whitney test). **(d)** Membrane resistance (Rm) (Ctrl: 114.2 ± 6.9 MΩ, n = 9 neurons; SynT: 115.8 ± 10.2 MΩ; n = 8, p = 0.90, two-tailed Student’s t test). **(e)** Membrane capacitance (Cm) (Ctrl: 227 ± 12 pF, n = 9 neurons; SynT: 197 ± 13 pF, n = 8; p = 0.105, two-tailed Student’s t test). **(f)** Representative miniature AMPA receptor-mediated postsynaptic currents (mEPSCs) from CA1 pyramidal cells voltage clamped at -70 mV in the presence of CPPene, picrotoxin and tetrodotoxin. Mean mEPSC amplitude was 17.1 ± 1.0 pA for control neurons (n = 4) and 14.2 ± 0.9 pA (n = 6) for SynTagMA-expressing neurons (p = 0.067, Mann Whitney test). Inter-event intervals were 667 ± 176 ms for control neurons (n = 4) and 1314 ± 553 ms (n = 6) for SynTagMA-expressing neurons (p = 0.48, Mann-Whitney test). **(g)** Sections of CA1 oblique dendrites expressing mCerulean alone (above) or mCerulean and SynTagMA (below). Scale bar is 5 µm. Mean spine density per 10 microns (± SEM) was 11.8 ± 1.2 for mCerulean-expressing neurons (7 neurons, 3 slices, 2896 spines) and 10.1 ± 0.7 for mCerulean and SynTagMA-expressing neurons (11 neurons, 5 slices, 3946 spines; p = 0.42, Mann-Whitney test).


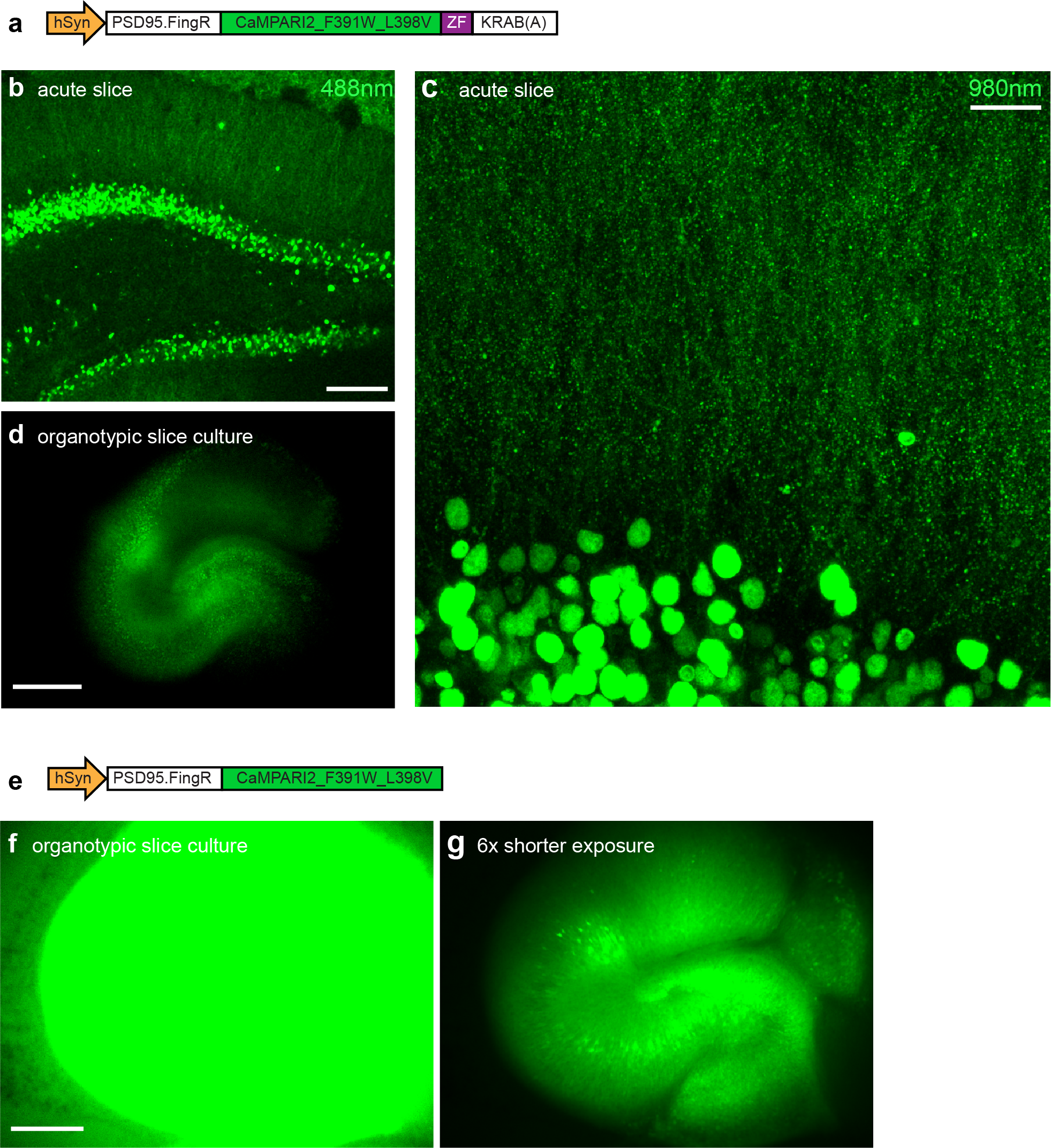


**Supplementary Figure 5: Effect of autoregulatory elements on postSynTagMA expression level. (a)** Scheme of postSynTagMA containing autoregulatory elements. **(b)** Confocal image of acute hippocampal slice from mouse expressing AAV9-postSynTagMA for two weeks. **(c)** Two-photon image (980 nm excitation) of dentate granule neurons expressing AAV9-postSynTagMA in an acute slice. Note dense, punctate expression in the molecular layers**. (d)** Organotypic rat hippocampal slice culture 4 days after AAV9-mediated transduction with postSynTagMA. **(e)** Scheme of postSynTagMA without autoregulatory elements. **(f)** Organotypic rat hippocampal slice culture 4 days after AAV9-mediated transduction with PSD.95-FingR_CaMPARI2_F391W_L398V (no ZF-KRAB). Virus titer and applied volume were matched to d). **(g)** Same culture, image was de-saturated by six-fold reduction in exposure time. Scale bars: 100 (b), 20 (c), 500 microns (d, f, g).

**
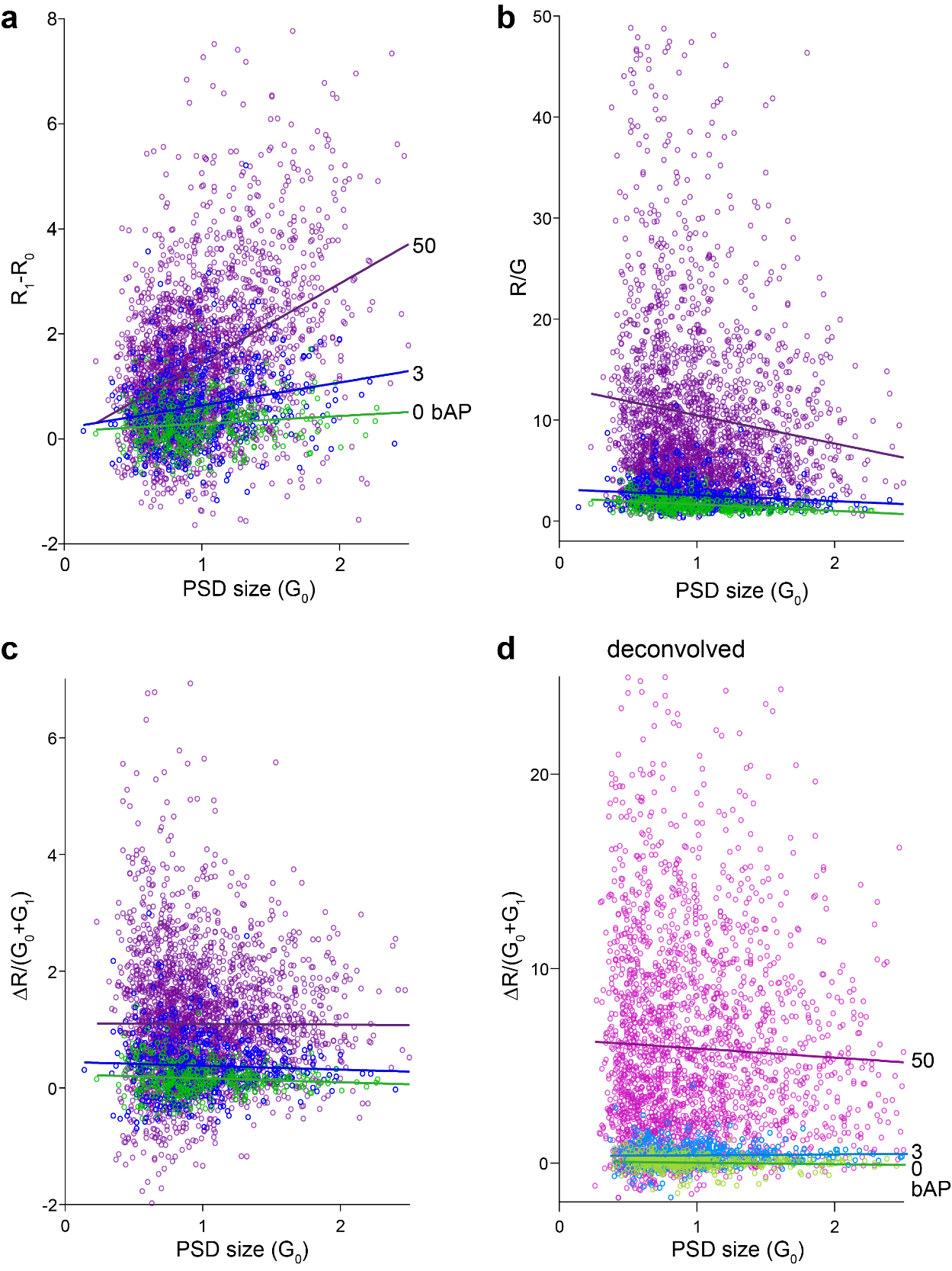
**

**Supplementary Figure 6: Comparison of conversion metrics vs synapse size (a)** Absolute change in red fluorescence (ΔR = R_1_ - R_0_) plotted against PSD size (G_0_). Slope of linear regression: 0.146 (0 bAP); 0.435 (3 bAP); 1.50 (50 bAP). Magenta: 50 bAPs. Blue: 3 bAPs. Green: 0 bAP. **(b)** Ratio after photoconversion (R_1_/G_1_) versus PSD size. Slope of linear regression: -0.63 (0 bAP); -0.59 (3 bAP); -2.8 (50 bAP). **(c)** ΔR/(G_1_ + G_0_) versus PSD size. Slope of linear regression: -0.068 (0 bAP); -0.066 (3 bAP); -0.011 (50 bAP). **(d)** Same analysis as in **c**), but on deconvolved data (AutoQuant X3). Note improved separation between stimulated and non-stimulated synapses. Pink: 50 bAPs. Light blue: 3 bAPs. Light green: 0 bAP.

**
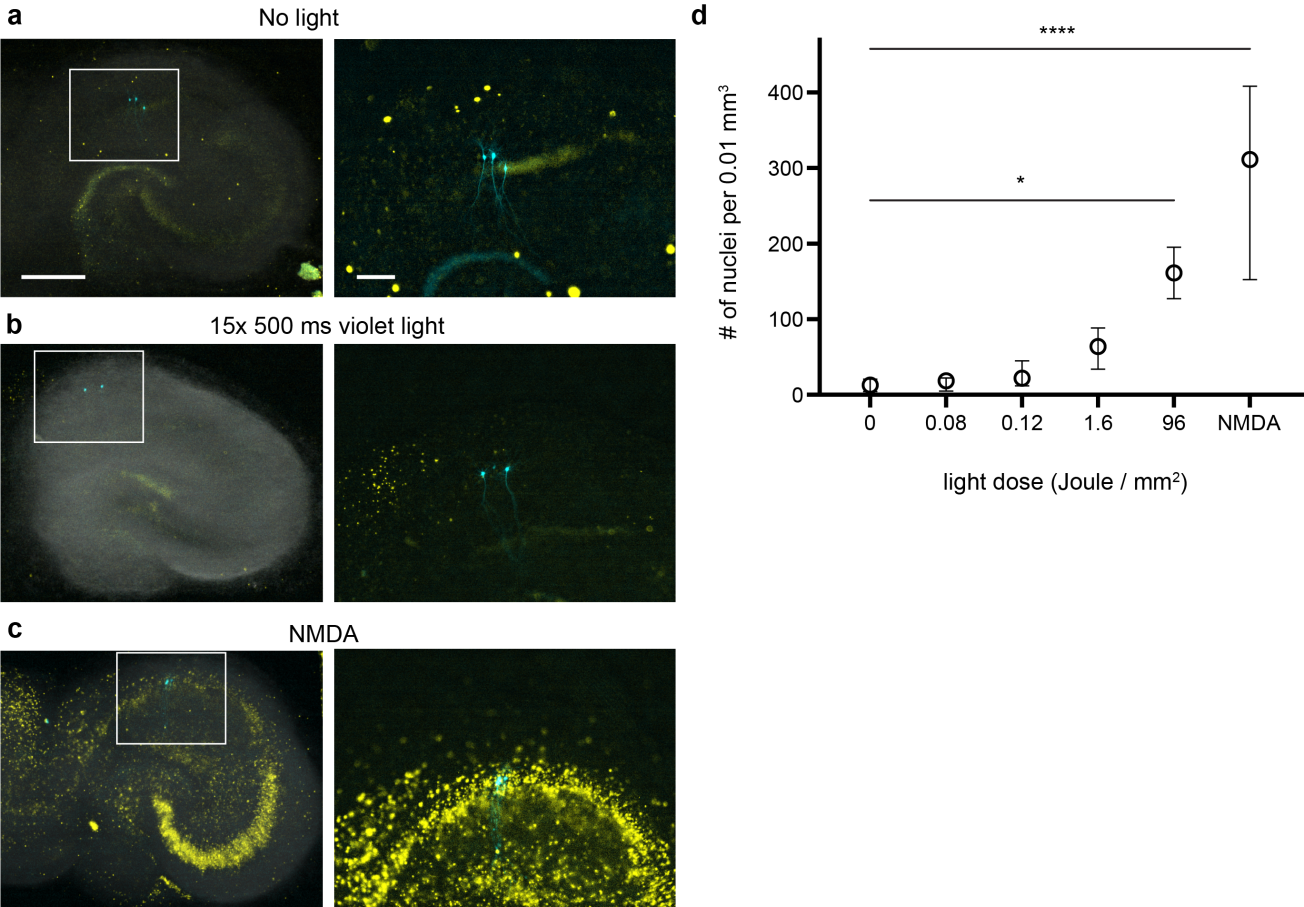
**

**Supplementary Figure 7: Assessing photodamage caused by violet light and NMDA in organotypic cultures by propidium iodide (PI) staining**. **(a-c)** Representative images of hippocampal slice cultures following incubation with propidium iodide, which enters and stains damaged cells. Overlay of Dodt-contrast image (grayscale), propidium iodide fluorescence (yellow) and cerulean-expressing CA1 pyramidal cells (cyan). Scale bars 500 µm and 200 µm, respectively. **(a)** Slice culture kept in the dark. **(b)** After exposure to our typical photoconversion protocol (15 x 500 ms light pulses, 395 nm, 16 mW mm^-2^) resulting in a cumulative light dose of 0.12 J mm^-2^. **(c)** After exposure to 1 mM NMDA for 1 hour. **(d)** Quantification of results vs light dose. Data are plotted as median and interquartile range. A one-way ANOVA followed by Dunnett’s multiple comparisons to the no light condition was used. No light (n = 5 slices) vs 0.08 J mm^-2^ (n = 3 slices, p > 0.999); No light vs 0.12 J mm^-2^ (n = 5 slices, p = 0.99); No light vs 1.6 J mm^-2^ (p = 0.74, n = 3 slices); No light vs 96 J mm^-2^ (*p < 0.0461, n = 2 slices); No light vs NMDA (**** p < 0.0001, n = 4 slices).


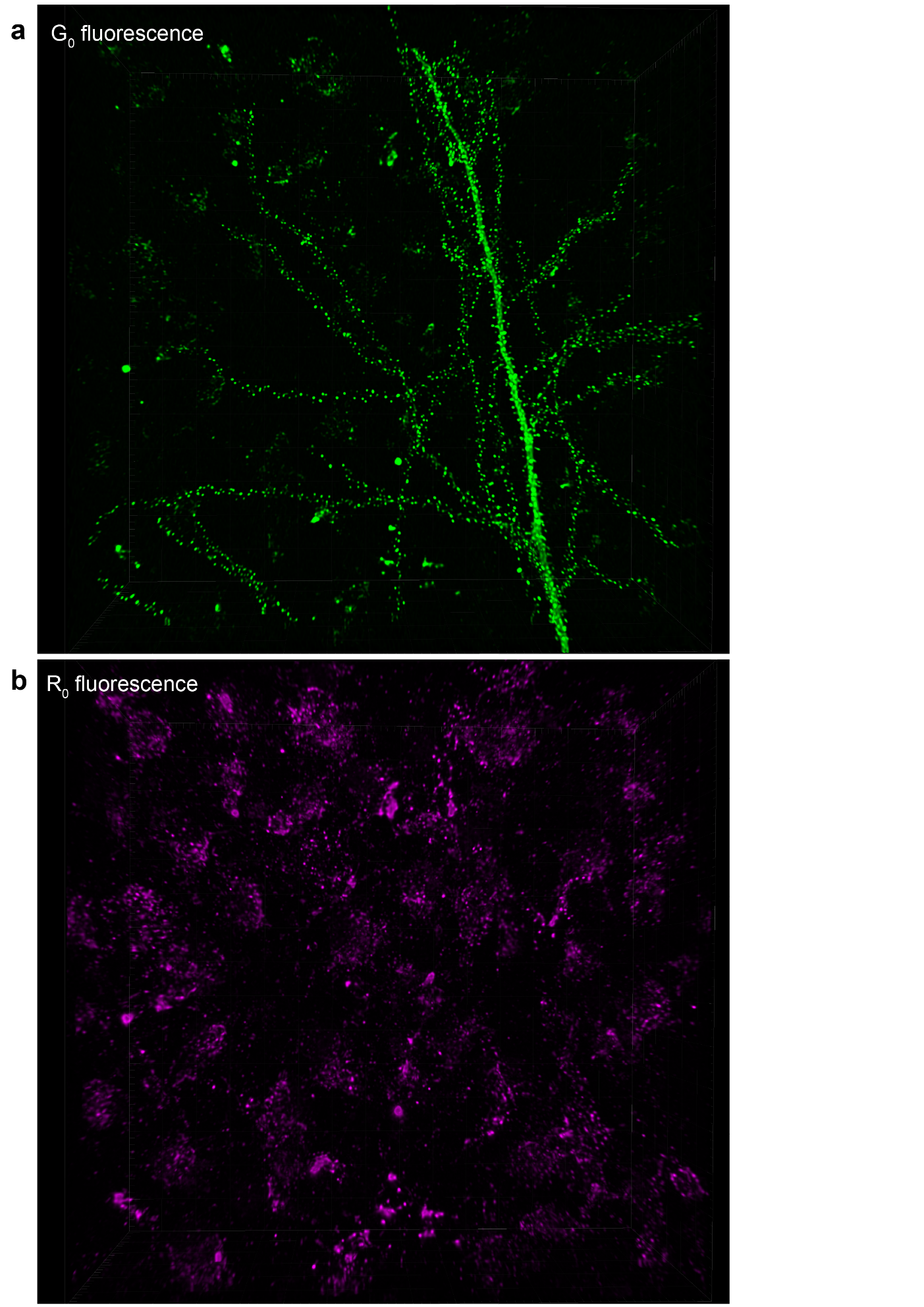


**
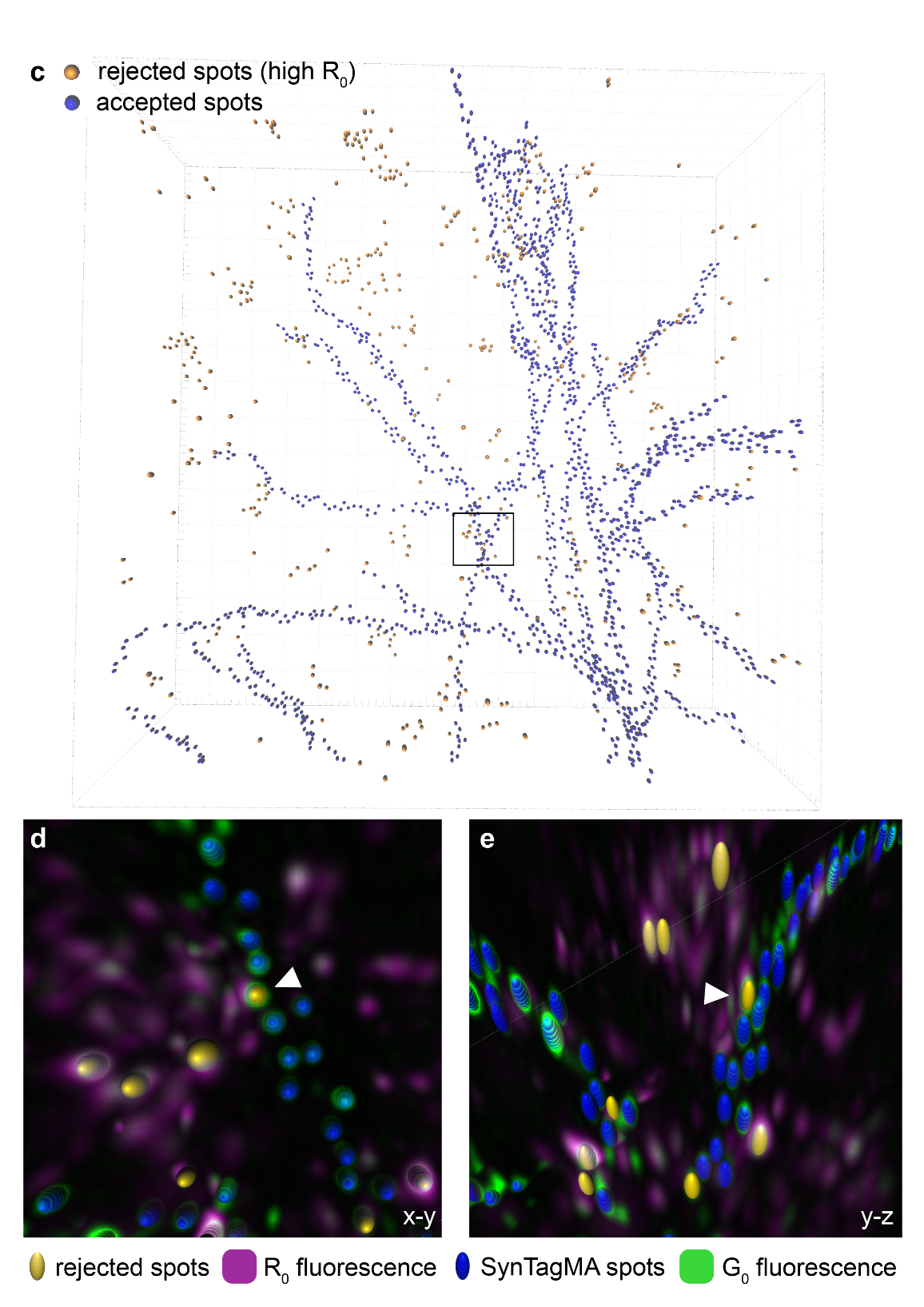
**

**Supplementary Figure 8: Exclusion of non-SynTagMA objects (spots) by their red fluorescence**. **(a)** SynTagMA-expressing pyramidal neuron before stimulation (G_0_). Dendritic branches in *stratum lacunosum-moleculare*, 980 nm excitation, 150 x 150 x 60 µm volume. **(b)** Corresponding view of red autofluorescence (R_0_) at 1040 nm excitation. **(c)** Spot detection in Imaris based on G_0_. Spots were subsequently classified according to their red fluorescence before simulation (R_0_). Spots with low R_0_ are SynTagMA-labeled spines (blue) while spots with high R_0_ (orange) were primarily detected in autofluorescent patches outside the labeled neuron. High R_0_ spots were rejected from further analysis. 3D images in Imaris (**a**-**b**), scale in **c**. **(d, e)** Zoomed-in views (rotated) of the area denoted by black square in a). White arrow points to a likely SynTagMA signal (green fluorescence) that due to its proximity to red autofluorescence has been rejected (yellow spot). Even in this problematic region, the large majority of auto-detected spots were sufficiently distant from autofluorescence to be analyzed (blue spots). Some small green spots escaped detection by the algorithm (no blue/yellow marker).

**
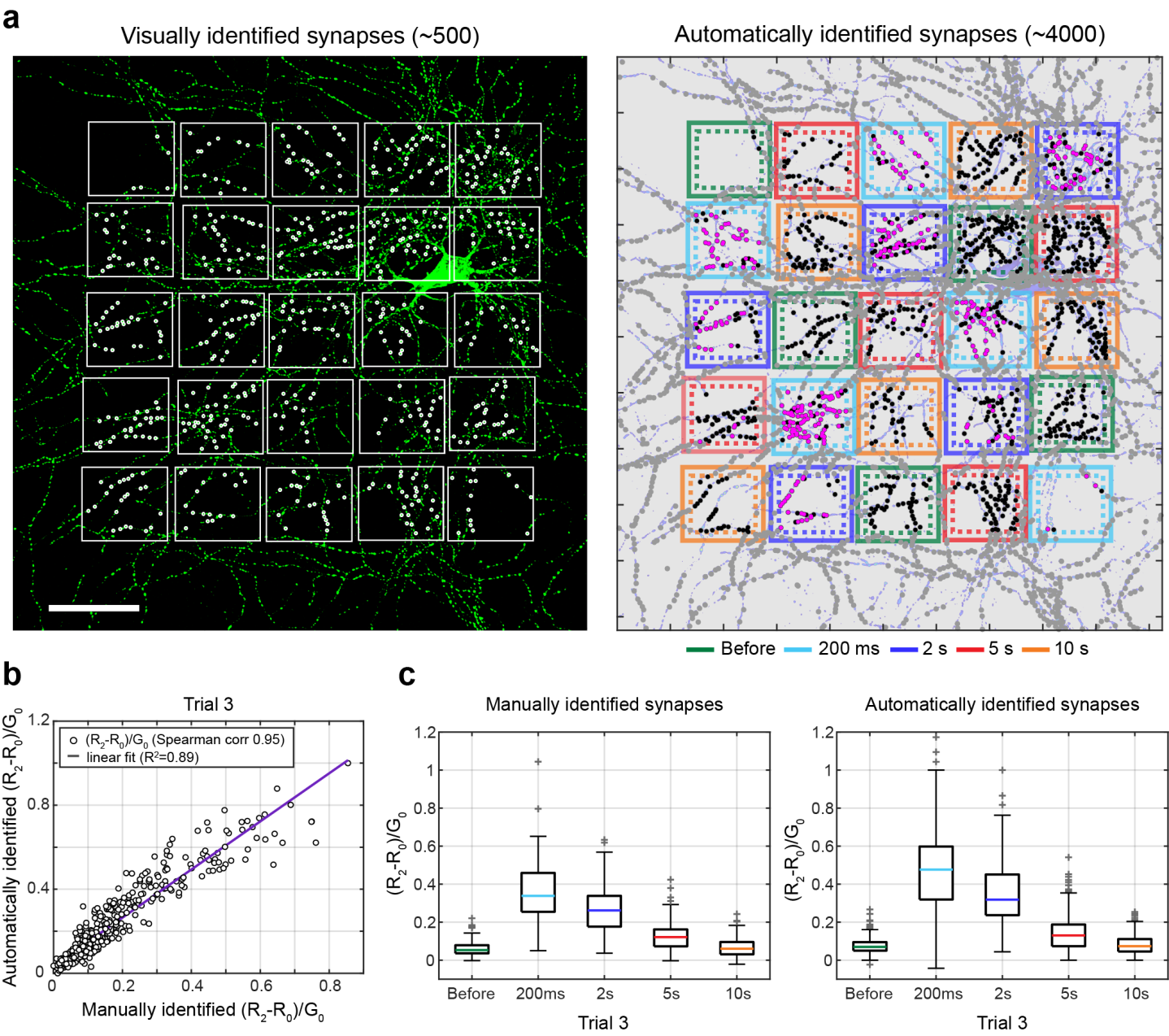
**

**Supplementary Figure 9: Comparison of analysis results with manual identification of ROIs vs analysis performed with SynapseLocator**. **(a)** Full-field view (maximum projection) of the same cultured hippocampal neuron expressing preSynTagMA as shown in Fig. 2 with overlaid photoconversion grid. Dashed lines are borders set for including automatically identified boutons in a group. Left: Regions of interest (~500 ROIs) were selected and manually curated using Fiji (white dots are ROIs). Right: Colored and grey dots indicate (ROIs) identified and analyzed by SynapseLocator. Grey spots were outside the illuminated fields and not quantified. Magenta spots: (R_2_ - R_0_)/G_0_ > 0.25. Black spots: (R_2_ - R_0_)/G_0_ < 0.25. Scale bar is 50 µm. **(b)** The photoconversion values from Trial 3 (see Fig. 2) from the group of boutons that were both manually and automatically identified plotted against each other (n = 413 boutons). **(c)** Photoconversion (R_2_ - R_0_)/G_0_ vs timing delay for the same data manually identified (right, n = 583 boutons) or identified and analyzed using SynapseLocator (left, n = 1059 boutons). Boxes: Median, 25% and 75% percentiles and whiskers the 1.5 interquartile interval. Grey + are outliers. The data distribution was not normal within any group (D’Agostino & Pearson test, P < 0.0001). A non-parametric Kruskal-Wallis test followed by Dunn’s multiple comparisons was used. Before manual vs before automatic (p = 0.46), 200 ms manual vs 200 ms automatic (p = 0.1184), 2 s manual vs 2 s automatic (p = 0.0503), 5 s manual vs 5 s automatic (p > 0.999), 10 s manual vs 10 s automatic (p = 0.3644).

**
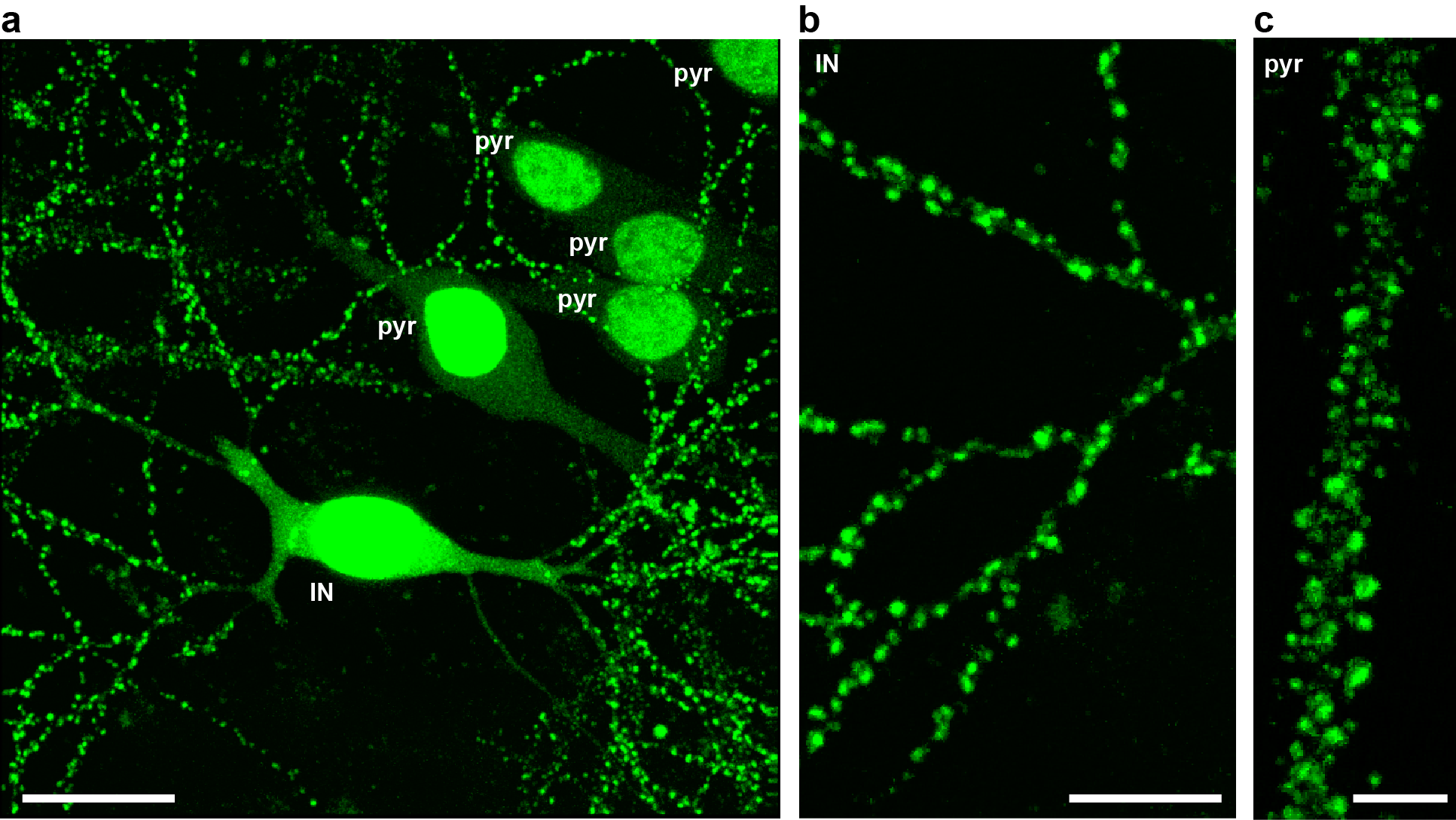
**

**Supplementary Figure 10. PostSynTagMA identifies excitatory synapses on interneurons**. **(a)** Two-photon image (maximum intensity projection) showing expression of postSynTagMA in an interneuron (IN) next to five pyramidal CA1 cells (pyr). Scale bar 20 µm. **(b)** Detail of postSynTagMA puncta in smooth interneuron dendrite (IN) and **(c)** spiny pyramidal neuron dendrite (pyr). Scale bars a) 20 µm b) 10 µm c) 5 µm.

**
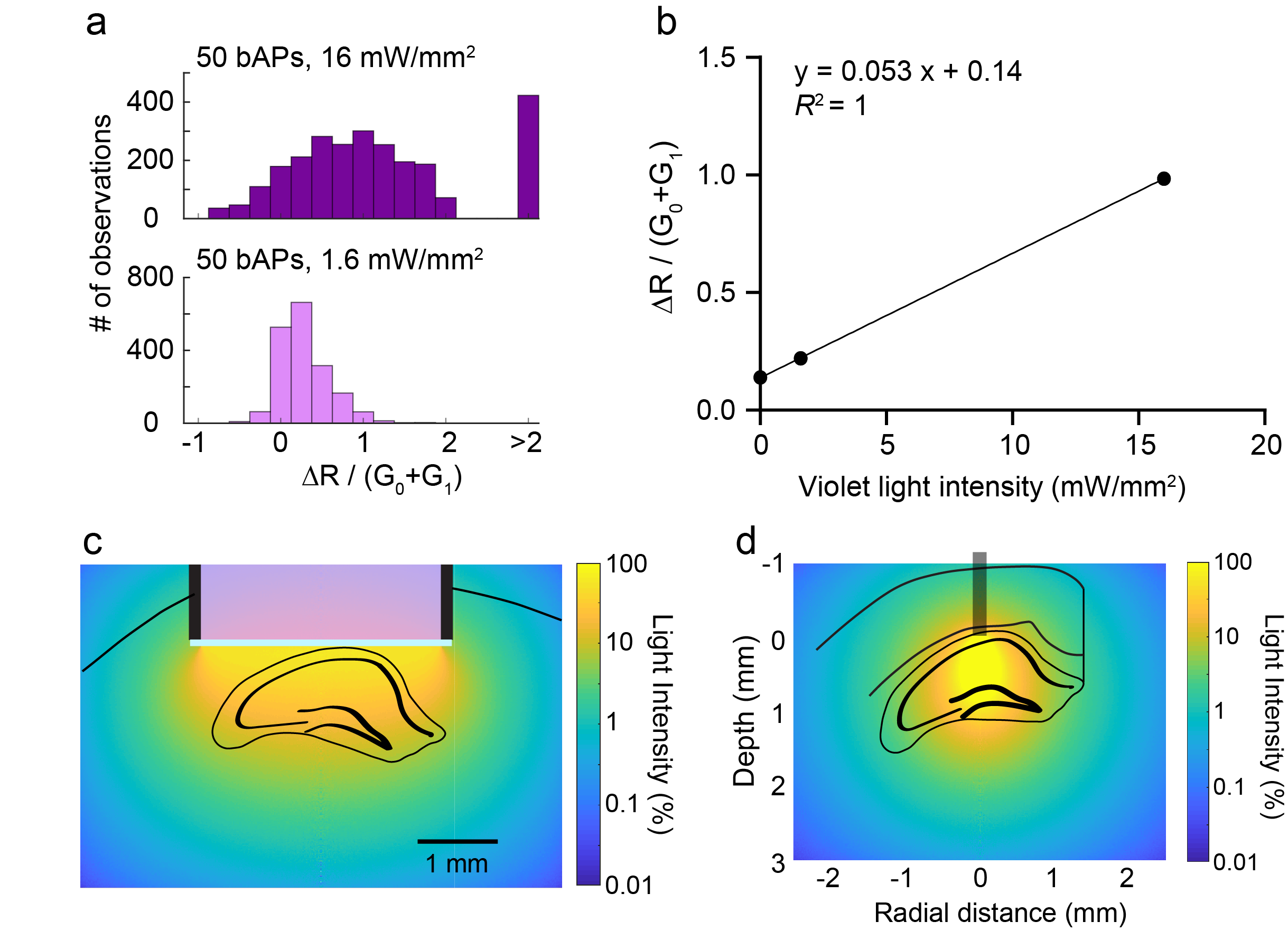
**

**Supplementary Figure 11. Intensity and depth-dependence of photoconversion. (a)** Histogram of photoconversion for 50 bAPs with 16 mW mm^-2^ (as in Fig. 4c) and 50 bAPs with 1.6 mW mm^-2^. **(b)** Photoconversion (ΔR/(G_0_+G_1_)) shows linear non-proportional relationship to violet light intensity. Median values taken from (a) with the 0 bAP condition shown in Fig. 4c. Black line represents the linear fit to the data. **(c)** Simulation of violet light illumination (405 nm) through a deep cranial window using an 0.8 NA objective (see Fig. 7). Note homogenous intensity throughout hippocampus for a given depth. **(d)** Simulation of violet light injection (10 mW) via 200 µm diameter light fiber with 0.22 NA. Depth attenuation is similar to the cranial window situation, but the intensity drops off more steeply with radial distance (xy).
