# Supplementary material for: Freeze-frame imaging of synaptic activity using SynTagMA
Source: Nat Commun. 2020 May 18;11:2464. doi: 10.1038/s41467-020-16315-4 (PMC7235013; doi:10.1038/s41467-020-16315-4)
Supplement: Supplementary file 3 — Reporting Summary [file 41467_2020_16315_MOESM3_ESM.pdf]

nature research

Corresponding author(s): Thomas Oertner, thomas.oertner@zmnh.uni-hamburg.de

Last updated by author(s): 2019/12/17

Reporting Summary

Nature Research wishes to improve the reproducibility of the work that we publish. This form provides structure for consistency and transparency in reporting. For further information on Nature Research policies, see Authors & Referees and the Editorial Policy Checklist.

Statistics

For all statistical analyses, confirm that the following items are present in the figure legend, table legend, main text, or Methods section.

n/a	Confirmed	
☐	☒	The exact sample size (n) for each experimental group/condition, given as a discrete number and unit of measurement
☐	☒	A statement on whether measurements were taken from distinct samples or whether the same sample was measured repeatedly
☐	☒	The statistical test(s) used AND whether they are one- or two-sided<br>*Only common tests should be described solely by name; describe more complex techniques in the Methods section.*
☒	☐	A description of all covariates tested
☐	☒	A description of any assumptions or corrections, such as tests of normality and adjustment for multiple comparisons
☐	☒	A full description of the statistical parameters including central tendency (e.g. means) or other basic estimates (e.g. regression coefficient) AND variation (e.g. standard deviation) or associated estimates of uncertainty (e.g. confidence intervals)
☐	☒	For null hypothesis testing, the test statistic (e.g. F, t, r) with confidence intervals, effect sizes, degrees of freedom and P value noted<br>*Give P values as exact values whenever suitable.*
☒	☐	For Bayesian analysis, information on the choice of priors and Markov chain Monte Carlo settings
☒	☐	For hierarchical and complex designs, identification of the appropriate level for tests and full reporting of outcomes
☐	☒	Estimates of effect sizes (e.g. Cohen's d, Pearson's r), indicating how they were calculated

Our web collection on statistics for biologists contains articles on many of the points above.

Software and code

Policy information about availability of computer code

Data collection	Imaging of dissociated culture used Pulse Pal (Open Ephys), Andor iQ software and Nikon elements for image acquisition. Two-photon imaging and electrophysiology data was acquired using the ScanImage (3.8 or 5.5) and Ephus or Wavesurfer Matlab-based suite of freeware.
Data analysis	For data analysis we used Fiji, Autoquant X3 (MediaCybernetics), Imaris (v.9.2 Bitplane AG), elastix, ANTs and Matlab (v2018b The Mathworks) softwares. The SynapseLocator program is deposited for use on GitHub (https://github.com/drchrisch/SynapseLocator). In vivo imaging analysis used suite2p (v0.7.1). Any additional code for analysis done in Matlab is available from the corresponding author on request. All statistics was done in either Matlab or Graphpad Prism v8.

For manuscripts utilizing custom algorithms or software that are central to the research but not yet described in published literature, software must be made available to editors/reviewers. We strongly encourage code deposition in a community repository (e.g. GitHub). See the Nature Research guidelines for submitting code & software for further information.

Data

Policy information about availability of data

All manuscripts must include a data availability statement. This statement should provide the following information, where applicable:
- Accession codes, unique identifiers, or web links for publicly available datasets
- A list of figures that have associated raw data
- A description of any restrictions on data availability

The datasets generated during and/or analysed during the current study are available from the corresponding author on request. Example datasets have been deposited on GitHub along with the analysis software (SynapseLocator) to extract numerical values.

Field-specific reporting

Please select the one below that is the best fit for your research. If you are not sure, read the appropriate sections before making your selection.

☒ Life sciences ☐ Behavioural & social sciences ☐ Ecological, evolutionary & environmental sciences

For a reference copy of the document with all sections, see nature.com/documents/nr-reporting-summary-flat.pdf

Life sciences study design

All studies must disclose on these points even when the disclosure is negative.

Sample size	No specific sample size calculations were done. We consider the synapse as an independent biological unit. In cases that the number of synapses analysed per neuron fell below 100, we included replications of more than one neuron/experiment.
Data exclusions	All neurons that were stimulated were analyzed. Automatically detected synapses were excluded from the analysis if red fluorescence was detected before stimulation. See Methods section for details.
Replication	Unless explictly stated, all experiments were replicated in at least 2 independent experiments on different days.
Randomization	In most experiments, our experimental units were neurons which were 'assigned' to an experiment by single-cell electroporation with synTagMA. At the time of stimulation/photoconversion, a transfected culture was taken out of the incubator and placed under the microscope. Randomization of cells/cultures with respect to treatments (e.g. stimulation) is therefore inherent to our experimental design.
Blinding	SynTagMA imaging experiments were analyzed automatically, using software that is by definition 'blind' to the experimental conditions. Blinding was used in this study for comparisons between cells expressing or not expressing SynTagMA (cell parameters, mEPSCs, spine density measurements). The researcher was only unblinded after all analyses were complete.

Reporting for specific materials, systems and methods

We require information from authors about some types of materials, experimental systems and methods used in many studies. Here, indicate whether each material, system or method listed is relevant to your study. If you are not sure if a list item applies to your research, read the appropriate section before selecting a response.

Materials & experimental systems

n/a	Involved in the study
☒	☐ Antibodies
☒	☐ Eukaryotic cell lines
☒	☐ Palaeontology
☐	☒ Animals and other organisms
☒	☐ Human research participants
☒	☐ Clinical data

Methods

n/a	Involved in the study
☒	☐ ChIP-seq
☒	☐ Flow cytometry
☒	☐ MRI-based neuroimaging

Animals and other organisms

Policy information about studies involving animals; ARRIVE guidelines recommended for reporting animal research

Laboratory animals	Wistar rats, provided by Janvier or Envigo, and Sprague-Dawley rats, provided by Charles River, of either sex were used to prepare organotypic and dissociated hippocampal cultures, respectively.<br>Male adult mice (C57BL/6J, provided by The Jackson Laboratory) were used for behavioral experiments.
Wild animals	The study did not involve wild animals.
Field-collected samples	The study did not involve field-collected samples.
Ethics oversight	Animal procedures for Wistar rats and C57BL/6 mice were approved by the Behörde für Gesundheit und Verbraucherschutz of the City of Hamburg, Germany. Procedures with Sprague-Dawley rats were approved by Dartmouth College's Institutional Animal Care and Use Committee (IACUC), Hanover, NH, USA.

Note that full information on the approval of the study protocol must also be provided in the manuscript.
