# Supplementary material for: Freeze-frame imaging of synaptic activity using SynTagMA
Source: Nat Commun. 2020 May 18;11:2464. doi: 10.1038/s41467-020-16315-4 (PMC7235013; doi:10.1038/s41467-020-16315-4)
Supplement: Supplementary file 4 — Description of Additional Supplementary Files [file 41467_2020_16315_MOESM4_ESM.pdf]

Description of Additional Supplementary Files

File Name: Supplementary Movie 1

Description: Spine in contact with Channelrhodopsin-expressing bouton becomes photoconverted. 3D video showing automated detection of synapses (silver objects) based on green fluorescence of postSynTagMA, red fluorescence inside the detected objects (masked), and zoom-in to a strongly photoconverted synapse (true positive) in contact with a presynaptic bouton (cyan). Note the large number of non-photoconverted synapses that are also distant from activated presynaptic terminals (true negative).

File Name: Supplementary Movie 2

Description: In vivo calcium imaging of CA1 neurons expressing GCaMP6f. Calcium imaging reveals similar patterns of activity during awake and under isoflurane anesthesia, but not under ketamine/xylazine anesthesia. Different conditions were tested on the same animal and CA1 area on different days. Ketamine/xylazine anesthesia reduced the number of active neurons and the intensity of calcium transients.

File Name: Supplementary Movie 3

Description: In vivo calcium imaging of CA1 neurons using postSynTagMA. Nuclear calcium elevations during treadmill running are visible as dimming of postSynTagMA green fluorescence. Total time: 4 min.
